# Tailored Personas of Online Health Information–Seeking Behaviors Among Men With Prostate Cancer Receiving Androgen Deprivation Therapy: Qualitative Study

**DOI:** 10.2196/90567

**Published:** 2026-07-20

**Authors:** Sijia Hou, Xiangyun Li, Ziyi Qi, Kun Li, Lu Chen, Keping Zhu, Wei Wang

**Affiliations:** 1 Department of Nursing, The First Affiliated Hospital, Zhejiang University School of Medicine, No.79, Qingchun Road, Shangcheng District, Hangzhou, Zhejiang, 310003, China, 86 13777826581; 2 School of Medicine, Zhejiang University, Hangzhou, Zhejiang, China; 3 Department of Nursing, Tianjin First Central Hospital, Tianjin, Tianjin, China; 4 Department of Nursing, The First Affiliated Hospital of Soochow University, Suzhou, Jiangsu, China

**Keywords:** prostate cancer, information-seeking behavior, digital health, patient persona, qualitative research

## Abstract

**Background:**

Patients with prostate cancer undergoing androgen deprivation therapy (ADT) must manage complex treatment side effects over extended periods outside the hospital, making online health information–seeking a key approach to self-management. However, individual differences in motivation, digital literacy, and psychosocial context significantly influence how patients seek and use online health information. The patient persona approach, which synthesizes individuals with similar behavioral patterns and needs into representative profiles, offers a practical method for capturing this heterogeneity and informing the design of tailored information support.

**Objective:**

This study aimed to explore the differences in online health information–seeking behavior among patients with prostate cancer undergoing ADT and construct patient personas to characterize distinct patterns of online health information–seeking experiences and related information support needs.

**Methods:**

A qualitative descriptive study was conducted in a tertiary hospital in Zhejiang Province, China, from July to October 2025. Purposive sampling was used to recruit patients receiving ADT. Semistructured interviews were conducted, and data were analyzed using inductive content analysis to derive codes, subcategories, and 4 core categories. Participants were systematically compared across these categories and grouped into personas based on recurring patterns. The personas were then presented with illustrative portraits.

**Results:**

A total of 20 participants were included. Four core categories were identified for persona construction: motivation, information access preferences, barriers, and needs. The personas were categorized as follows: patients demonstrating proactive information management and high health literacy, patients exhibiting avoidant information-seeking driven by latent anxiety, patients engaging in family-doctor–mediated information-seeking, and patients displaying dependent and passive information-seeking behavior.

**Conclusions:**

Online health information–seeking behavior among patients with prostate cancer receiving ADT is highly heterogeneous and shaped by individual capability, emotional responses, and sociocultural factors. The identified personas may provide a preliminary and practice-oriented framework for developing more differentiated, culturally sensitive, and patient-centered online health information support strategies for patients receiving long-term ADT.

## Introduction

Prostate cancer is one of the most common malignant tumors among men worldwide and is closely linked to androgen [1]. Androgen deprivation therapy (ADT) remains a cornerstone treatment for advanced or high-risk prostate cancer [2]. Although ADT effectively delays disease progression, patients often experience persistent and distressing side effects, including hot flashes, fatigue, sexual dysfunction, metabolic disorders, and psychological disturbances [3]. Because ADT is typically administered over several years, patients must manage fluctuating symptoms, treatment uncertainty, and lifestyle adjustments largely outside hospital settings, making timely access to health information essential for long-term self-management [4].

Online health information–seeking (OHIS) has become an increasingly important resource for patients with cancer [5]. Online health information–seeking behavior (OHISB) refers to the process by which individuals use the internet to satisfy informational needs, reduce uncertainty, and support health management [6]. Studies indicate that approximately 70% to 80% of patients with cancer have searched for disease-related information online [7], and doing so may improve disease knowledge, enhance self-efficacy, and facilitate participation in medical decision-making [8,9]. For patients receiving ADT, OHIS may be particularly important. Prostate cancer primarily affects middle-aged and older men, groups that increasingly engage with digital media but vary substantially in digital literacy and technology confidence [10]. In addition, ADT is usually delivered over prolonged periods in home settings, requiring patients to manage side effects and follow complex care plans between outpatient visits [11]. Furthermore, prostate cancer involves sensitive issues related to sexual function, urination, and masculinity. The relative anonymity of online environments may therefore provide a psychologically safer space for men to seek information and communicate with peers [12]. These characteristics suggest considerable heterogeneity in how patients engage with online health information during ADT.

Despite its potential benefits, online information-seeking also presents important challenges. Cancer-related online resources are often fragmented, inconsistent in quality, and poorly matched to patients’ evolving informational needs [5,13]. Patients also differ substantially in their motivations for seeking information, digital literacy, emotional coping styles, trust in online resources, and ability to integrate information into decision-making [7,10,14]. Consequently, some patients may benefit from online resources, whereas others may experience confusion, information overload, anxiety, or avoidance. Existing studies have primarily examined OHISB through individual factors such as age, education, health literacy, or information needs, providing limited insight into the lived experiences and diverse patterns of information engagement among patients receiving long-term treatment management [5]. Although qualitative studies have begun to explore patients’ informational experiences, research specifically examining the OHIS experiences of patients with prostate cancer receiving long-term ADT remains limited [15]. This gap is particularly important because patients’ information needs and coping behaviors may evolve throughout long-term treatment and be influenced by masculinity-related concerns and cultural context. As a result, current digital health support systems may have limited ability to address patients’ evolving and individualized informational needs [16].

Under the framework of user-centered design, recognizing meaningful differences in patients’ behaviors and support needs is essential for developing effective information support interventions [17]. The persona approach is an empirically grounded method that synthesizes individuals sharing similar characteristics and behavioral patterns into representative typologies [18,19]. Personas can be developed using qualitative, quantitative, or mixed methods. In exploratory health care research, qualitative approaches are particularly valuable because they allow complex patient experiences and behavioral patterns to emerge inductively from patients’ lived narratives and be translated into clinically meaningful profiles [20]. In health care research, qualitative persona approaches have been applied in chronic disease management and digital health design, including cardiac rehabilitation self-management [21], mHealth interventions targeting medication adherence [22], personalized mental health service design for older adults [23], and the characterization of symptom management preferences and information needs among patients with cancer [24-26]. These studies suggest that persona-informed approaches can translate complex patient experiences into actionable representations for intervention design and may improve the precision of health information delivery, support treatment adherence [27], and enhance care experiences [28]. Given the evolving informational demands and diverse contextual influences experienced during long-term ADT, the persona approach may provide a particularly useful framework for understanding different patterns of online health information engagement and informing more tailored information support strategies.

Despite increasing attention to OHISB among patients with cancer, research specifically focusing on men receiving long-term ADT remains limited, and existing evidence provides limited guidance for translating patient heterogeneity into practical information support strategies. A persona approach may offer a useful framework for synthesizing recurring patterns of patient experiences and support needs into clinically meaningful profiles. Accordingly, this study aimed to apply a qualitative persona approach to systematically characterize the heterogeneous OHISB of patients with prostate cancer undergoing ADT and to develop patient personas that may inform the design of more differentiated and patient-centered online health information support.

## Methods

### Design

This study used a descriptive qualitative design to explore the OHIS experiences of patients with prostate cancer receiving ADT. This approach is particularly suitable for obtaining practice-oriented and experience-near accounts of participants’ perspectives in naturalistic clinical contexts [29]. The study followed the Standards for Reporting Qualitative Research (SRQR) guidelines (Checklist 1) [30].

### Researcher Characteristics and Reflexivity

The research team included nurses with clinical experience in urological oncology: 3 researchers, 2 research assistants, and 2 clinical experts. The lead researcher (SH) had 3 years of clinical experience in a urology outpatient clinic and conducted all interviews within that setting. This insider positionality facilitated rapport with participants but also risked shaping data interpretation through pre-existing clinical assumptions. To manage this influence, the research team maintained reflexive awareness throughout data collection and analysis by using reflexive journals after interviews, conducting regular team discussions to critically examine emerging interpretations, and comparing independent coding perspectives across researchers with different professional roles. All team members had prior training in qualitative research methods.

### Setting and Participants

This study was conducted from July to October 2025 in a tertiary hospital in Zhejiang Province, China. The hospital performs approximately 3000 prostate biopsies each year, ensuring a sufficient patient population for the study.

Participants were recruited using purposive sampling to ensure variation in age, education level, treatment duration, and health literacy. Inclusion criteria were as follows: (1) diagnosed with prostate cancer, (2) receiving ADT for ≥3 months, (3) aged 18 years or older, (4) able to communicate in Mandarin, and (5) willing to participate and provide informed consent. Exclusion criteria were cognitive impairment, severe mental illness, or other serious diseases that hindered participation (such as concurrent other malignant tumors or severe complications).

Participants were recruited from the urology outpatient clinic and inpatient department of the study hospital. Potentially eligible participants were first identified by the lead researcher (SH) through the hospital’s electronic medical record system according to the inclusion criteria. After confirming eligibility with the treating urologists in the outpatient clinic or the responsible nurses in the inpatient department, patients were introduced to the study by their clinician or nurse. Those who expressed interest were subsequently approached by the lead researcher, who explained the study purpose and procedures, reconfirmed eligibility, and obtained written informed consent before the interview.

The number of participants was determined according to the data saturation principle [31]. Saturation was assessed iteratively throughout data collection and analysis. After each interview, emerging participant characteristics were compared with the developing persona framework to identify features that were insufficiently represented or not adequately captured by existing persona patterns, consistent with a process of negative case analysis. By the 18th interview, no substantively new characteristic patterns relevant to persona construction were identified, and the existing framework was considered sufficient to capture the range of OHISB observed across the sample. Two additional interviews were subsequently conducted to confirm the adequacy of the framework. As no new persona characteristics or participant patterns emerged, a final sample of 20 participants was considered sufficient to support the identification of 4 representative personas.

### Data Collection

Following written informed consent, basic demographic and clinical information, including age, education level, and treatment duration, was collected. Interviews were conducted in a quiet, private meeting room, except for 3 participants who opted for telephone interviews, and were generally scheduled immediately after outpatient consultations or, for inpatients, before hospital discharge. Each interview was conducted by 2 researchers (SH and ZQ), with one leading the interview and the other taking field notes and observing nonverbal cues. Interviews lasted approximately 30 to 60 minutes. With participants’ permission, all interviews were audio-recorded and transcribed verbatim within 24 hours. During the interviews, nonverbal information such as expressions, tone, and gestures of the participants was recorded, and timely follow-up questions and clarifications were made based on the participants’ statements.

A semistructured interview guide was developed based on the study objectives and refined through discussion with 1 urology nursing expert and 2 urology clinicians. Two pilot interviews were conducted to assess the clarity and appropriateness of the interview questions. Data from the pilot interviews were not included in the final analysis. The final interview guide is provided in Multimedia Appendix 1.

### Data Analysis

This study used an inductive content analysis approach to analyze the interview data [32]. This approach was selected because no existing theoretical framework adequately captured the heterogeneity of OHISB among patients with prostate cancer receiving ADT. Inductive analysis allowed participant characteristics and behavioral patterns to emerge directly from the data and subsequently informed persona development. MAXQDA (version 2020; VERBI Software) was used for data management and coding.

### Coding and Category Development

The analysis proceeded through 3 iterative stages. Stage 1 is open coding. The unit of analysis was the meaning unit, defined as words, sentences, or paragraphs containing a coherent idea related to OHISB. Two researchers (SH and XL) independently read the transcripts multiple times to achieve immersion in the data. Meaning units were identified and assigned initial codes that reflected their underlying meaning. Stage 2 is the formation of subcategories. The 2 researchers met weekly to compare independently generated codes and discuss similarities and differences in interpretation. Through constant comparison, conceptually related codes were grouped into more abstract subcategories. Stage 3 is abstraction of higher-order categories. Subcategories were further compared and synthesized through iterative team discussions. Through abstraction and constant comparison, 4 higher-order categories were identified that captured recurring patterns in participants’ OHIS experiences. These categories subsequently served as the analytical domains for persona construction. The detailed codebook is provided in Multimedia Appendix 2.

### Persona Development Process

Persona construction was informed by user-centered persona approaches commonly used in health informatics research, in which representative archetypes are developed through systematic comparison of shared behavioral characteristics [18,19]. After coding, participant characteristics identified across the 4 analytical categories were summarized into a participant characteristic matrix (Multimedia Appendix 3). Participants were then systematically compared across this matrix to identify recurring patterns of similarity and difference. Participants sharing similar combinations of motivations, information-seeking preferences, barriers, and support needs were iteratively grouped and compared. Through this iterative comparison process, 4 representative personas reflecting distinct patterns of OHISB were identified.

Each persona was named according to its dominant behavioral characteristics. Narrative persona descriptions integrated the defining motivations, preferences, barriers, and support needs associated with each persona type. Illustrative portrait images were AI-generated based on synthesized textual descriptions to support the visual communication of generalized persona characteristics rather than represent identifiable individuals.

### Rigor

To enhance trustworthiness and analytic rigor, several strategies were used throughout the study. Investigator triangulation was achieved through independent coding by 2 researchers, followed by regular comparison and discussion of interpretations. When disagreements could not be resolved through discussion between the 2 primary coders, a third senior researcher (WW) was consulted. The third researcher independently reviewed the relevant data segments and the codes in question, and a final decision was reached through team discussion until consensus was achieved. Participant validation and expert review were additionally used to strengthen the credibility and interpretive resonance of the findings.

Before participant validation, 2 clinical experts (an oncology head nurse and a urologist) reviewed the preliminary personas. They evaluated whether the personas reflected clinically recognizable patterns and whether the identified characteristics, barriers, and support needs aligned with their clinical observations. Based on their feedback, descriptions of digital literacy and information-seeking frequency were further clarified in Multimedia Appendix 3. Any discrepancies between expert feedback and the research team’s interpretations were resolved through joint re-examination of the relevant data and iterative discussion within the research team.

All 20 participants were invited to participate in member checking, and 17 responded. Validation occurred in 2 stages. First, preliminary analytical categories were shared with participants. Second, after persona construction, participants reviewed summary descriptions of the personas and were asked whether the personas reflected their experiences. Feedback was collected through outpatient conversations and WeChat. Fifteen participants considered the personas to meaningfully reflect their experiences, whereas 2 suggested minor revisions that were incorporated into the final descriptions (eg, incorporating descriptions of family dynamics, expanding emotional support needs, and softening negative characteristics in one persona type). This process constituted member checking of interpretations rather than factual verification of quotations, which had already been confirmed during the interviews.

### Ethical Considerations

This research was approved by the Clinical Research Ethics Committee of the First Affiliated Hospital of Zhejiang University School of Medicine (approval number 2025‐0202). All participants signed informed consent forms before participation. Each participant was assigned a unique code (eg, P1-P20) to ensure anonymity. Throughout the research process, the information and privacy of the participants were strictly confidential. All recordings were used for research purposes only and were not disclosed or used for other purposes without the consent of the participants. As compensation for their time and contribution, each participant received a small gift (valued at approximately RMB 20‐50) and was offered a brief disease-related consultation by the clinical team following the interview.

## Results

### Participant Characteristics

A total of 20 eligible patients receiving ADT participated in this study (Table 1). Participants ranged in age from 57 to 89 years, with a mean age of 70.5 (SD 9.4) years. The duration of ADT ranged from 3 to 65 months, with a mean duration of 21.4 (SD 19.1) months. Fourteen participants received ADT combined with radical prostatectomy, whereas 6 received ADT alone.

**Table 1.** Characteristics of the participants.

ID	Age (years)	Education	Occupation	Marital status	Residence	ADT^a^ duration (months)	Treatments	Medication
P1	62	Bachelor’s degree	Retired	Married	Urban area	41	RP^b^+ADT	LHRH^c^ agonist
P2	62	High school	Self-employed	Married	Rural area	18	RP+ADT	LHRH agonist+antiandrogens
P3	70	Primary school	Self-employed	Never married	Rural area	4	ADT	LHRH agonist+antiandrogen
P4	78	Associate’s degree	Retired	Married	Urban area	16	RP+ADT	LHRH agonist
P5	66	High school	Self-employed	Married	Rural area	6	ADT+RP	LHRH agonist
P6	72	Middle school	Retired	Married	Urban area	7	ADT+RP	LHRH agonist+antiandrogen
P7	73	High school	Retired	Married	Urban area	12	ADT+RP	LHRH agonist
P8	61	High school	Retired	Married	Urban area	8	ADT+RP	LHRH agonist+antiandrogen
P9	66	High school	Self-employed	Married	Urban area	20	RP+ADT	LHRH agonist+antiandrogen
P10	74	Middle school	Retired	Married	Rural area	42	ADT	LHRH agonist
P11	77	Associate’s degree	Retired	Married	Rural area	65	RP+ADT	LHRH agonist
P12	78	High school	Retired	Married	Rural area	3	ADT	LHRH antagonist+antiandrogen
P13	57	High school	Employed	Married	Urban area	10	ADT+RP	LHRH agonist+antiandrogen
P14	89	Bachelor’s degree	Retired	Married	Urban area	6	ADT	LHRH agonist+antiandrogen
P15	57	Middle school	Self-employed	Married	Rural area	3	ADT	LHRH antagonist+antiandrogen
P16	86	Middle school	Retired	Married	Urban area	35	ADT	LHRH antagonist+antiandrogen
P17	64	Associate’s degree	Retired	Married	Urban area	32	RP+ADT	LHRH agonist+antiandrogen
P18	84	High school	Retired	Married	Urban area	63	RP+ADT	LHRH agonist+androgen synthesis inhibitor
P19	71	Associate’s degree	Employed	Married	Urban area	12	RP+ADT	LHRH agonist+antiandrogen
P20	63	Associate’s degree	Retired	Married	Urban area	24	RP+ADT	LHRH agonist+antiandrogen

a ADT: androgen deprivation therapy.

b RP: radical prostatectomy.

c LHRH: luteinizing hormone-releasing hormone.

### Tailored Persona of Online Health Information–Seeking Behavior in Patients Receiving ADT

Based on recurring patterns across motivations, information access preferences, barriers, and support needs, 4 personas of OHISB were identified among patients receiving ADT. Table 2 presents an illustrative example of how participant characteristics were synthesized into a persona type, and Table 3 summarizes the defining characteristics of the 4 identified personas. Illustrative portraits were included to support visualization of the personas and do not represent actual participants.

**Table 2.** Illustrative example of participant grouping and persona synthesis for persona A.^a^

Participant	Characteristic patterns across 4 categories				Shared characteristics contributing to grouping
	Motivation	Information access preference	Barrier	Need	
P1	Seeking comparative treatment knowledge; maintaining sense of control through information	General internet platforms; physician-endorsed platforms	Information overload; contradictory content	Structured stage-specific information; direct physician communication	Proactive, self-directed information-seeking; persistent engagement with online health information; strong desire for structured, verified content
P2	Seeking treatment rationale and prognosis; peer validation	General internet platforms; online patient communities	Information fragmentation; inconsistent quality	Direct physician communication channel; structured peer support	High initiative in information acquisition; reliance on authoritative sources; active self-management orientation
P7	Seeking treatment process clarity; bridging outpatient communication gaps	General internet platforms; physician-endorsed platforms	Information inconsistency; distress from negative content	Structured stage-specific information; emotionally neutral content	Consistent active information engagement; focus on treatment clarity; preference for institutional credibility
P8	Seeking treatment process clarity; maintaining sense of control through information	General internet platforms; online patient communities	Information overload; contradictory content, online fees expensive	Symptom management guidance; structured treatment information	Proactive information-seeking to enhance control; active multiplatform use; tolerance for barriers despite frustration
P13	Seeking treatment rationale; supplementing outpatient information	Physician-endorsed platforms; medical consultation platforms	Limited consultation time; information fragmentation	Structured information with visuals; institutionally verified platforms	Purpose-driven information-seeking; emphasis on authority and accuracy; high digital self-efficacy
P19	Seeking treatment process clarity; supplementing clinical explanations	Physician-endorsed platforms; direct physician communication	Insufficient authoritative and easily understandable information on the internet	Pretreatment ADT^b^ education; stage-specific explanations	Sustained information engagement; focus on treatment mechanisms; active participation in care decisions

a Through continuous comparison of the main characteristics of 20 participants in 4 categories, the above 6 participants showed a highly consistent pattern: prominent initiative, active multiplatform use, frustration with the quality of information (despite these obstacles, they continue to seek information), and the need for structured information. Compared with other participants, these 6 are younger, more highly educated, and report higher digital literacy and seeking frequency (Table 1 and Multimedia Appendix 3). They represent a highly healthy literacy group. Based on these shared characteristics, we grouped these participants together and subsequently named the group persona A: patients demonstrating proactive information management and high health literacy.

b ADT: androgen deprivation therapy.

**Table 3.** Tailored persona of online health information–seeking behavior (OHISB) in patients receiving androgen deprivation therapy (ADT).

Characteristics	Persona A	Persona B	Persona C	Persona D
Name	Patients demonstrating proactive information management and high health literacy	Patients exhibiting avoidant information-seeking driven by latent anxiety	Patients engaging in family-doctor–mediated information-seeking	Patients displaying dependent and passive information–seeking behavior
Portrait	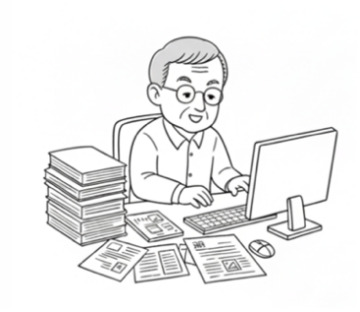	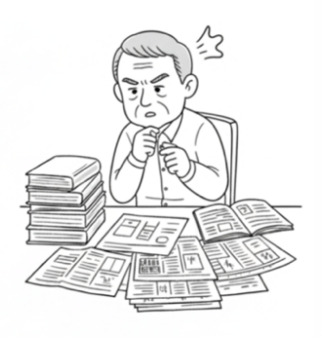	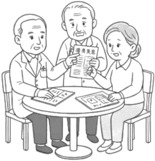	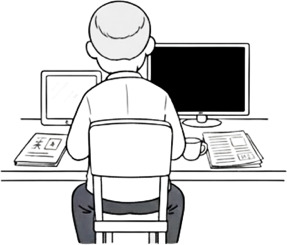
Participant	P1, P2, P7, P8, P13, P19	P6, P9, P10, P11, P16, P17	P5, P12, P14, P15, P20	P3, P4, P18
Age (years)	57‐73	64‐86	57‐89	70‐84
Education	High school to bachelor’s	Middle to associate’s	Middle to bachelor’s	Primary to associate’s
ADT^a^ duration (months)	8‐41	7‐65	3‐24	4‐63
Digital literacy	High	High/medium	Medium/low	Very low
Seeking frequency	High	High (sudden)	Medium/low	Low
Key motivations	Control disease progression Gain comprehensive knowledge Decision autonomy	Alleviate anxiety Manage symptoms Distinguish progression Maintain male identity integrity	Reduce cognitive burden Maintain family roles Ensure correct adherence	Trust health care authority Avoid information overload and anxiety Maintain a stable daily life
Main barriers	Information overload Fragmented or inconsistent information Quality variance	Unreliable content Emotional avoidance Stigma and privacy concerns	Limited digital skills Family overprotection Difficulty in understanding medical information	Limited digital access Low health literacy Fatalism beliefs
Primary support needs	Evidence-based treatment information Personalized self-management guidance Expert consultation support	Psychological support and symptom management guidance Gradual and supportive information delivery	Family-inclusive information support Shared follow-up and medication reminders Caregiver education resources	Simplified educational materials Offline reminder support Easy-to-understand instructions
Preferred sources	Professional websites; physician-endorsed platforms; patient communities	Search engines; social media; online peer communities	Medical consultation; family-assisted sources; health apps	Offline; face-to-face communication; printed or broadcast materials

a ADT: androgen deprivation therapy.

### Persona A: Patients Demonstrating Proactive Information Management and High Health Literacy

These patients were mainly middle-aged and older men aged 57 to 73 years, who were relatively young prostate cancer survivors. They typically had a moderate or high level of education, good digital literacy, and tended to be proactive in disease management. Most of these patients were in the early-to-mid stages of the disease, emphasizing the use of information access to support decision-making and optimize communication with doctors. From the early stages of diagnosis, patients sought information about treatment options, medication differences, and side effect mechanisms while continuing to self-monitor.

Motivations: Their primary motivation was to reduce uncertainty and maintain a sense of control over the disease trajectory. They hoped to better understand treatment processes and participate more actively in decision-making and long-term management.

I want to figure out the whole treatment process so that I know what will happen next.

[P8]

If I can understand the whole procedure, such as why hormone treatment is before or after surgery, and what the long-term side effects are, I can better cooperate with the treatment.

[P19]

Barriers: Despite their active engagement, these patients frequently experienced information overload and fragmented content. They often had to compare information across multiple platforms and independently evaluate the credibility of different sources.

The information on the Internet is too scattered, and there are too many popular science accounts on TikTok and Xiaohongshu now. I can’t be sure which one is right.

[P2]

There are different statements in different places. For example, it is difficult to judge the long-term impact of side effects.

[P1]

Needs: These patients preferred well-structured and authoritative online information platforms covering treatment processes, recurrence and metastasis, symptom management, medication differences, and long-term monitoring. They emphasized the importance of platform credibility and expressed a strong preference for hospital-recommended information sources and functions such as symptom tracking and prostate-specific antigen (PSA) monitoring.

If the common problems can be sorted out by category, I don’t have to piece them together.

[P7]

In my opinion, if someone wants to get information from the Internet, it is at least the responsibility of hospitals to recommend authoritative websites to patients to make sure they know what is right and what is wrong.

[P13]

It’s hard to see a doctor now. I wish I could communicate with the doctor on WeChat. It’s better to record my PSA level and the symptoms after taking the medicine.

[P2]

### Persona B: Patients Exhibiting Avoidant Information-Seeking Behavior Driven by Latent Anxiety

These patients varied in age and educational background and generally demonstrated moderate digital literacy. They usually paid moderate attention to their health conditions and tended to avoid sensitive health information related to topics such as prognosis and changes in male characteristics. However, when sudden or worsening side effects of ADT (such as hot flashes and joint pain) affected their daily lives or their sense of male identity, it triggered anxiety in a short period of time, leading to an intensive and short-term online search state.

Motivations: Their information-seeking was mainly triggered by symptom-related uncertainty. They were eager to determine whether their symptoms were severe and how to relieve them quickly to restore a sense of stability in their daily lives.

After switching to a new medication, I experienced muscle soreness and joint pain. I’m not sure whether it’s side effects or disease progression. I have been looking for ways to alleviate it.

[P9]

I sweated at night, my mouth was dry, and my back hurt. I don’t know if it was metastasis or a side effect, so I went to the QQ group to ask, and the patient said it was a medication reaction.

[P11]

Barriers: These patients were highly sensitive to frightening or conflicting online information, which often increased anxiety and led them to discontinue searching altogether. They also tended to avoid discussing sensitive issues, such as breast enlargement or sexual dysfunction, directly with health care professionals or family members.

I do worry...my breasts have grown bigger. It’s actually a burden for me. But you won’t ask me, so I won’t mention it on my own.

[P17]

The language on the Internet, you know, they write very scary in order to attract attention, and I don’t dare to look down when I see it. What “beware of these symptoms” and “late signals,” I didn’t dare to look down when I saw the title.

[P16]

Needs: These patients preferred concise, emotionally supportive, and easy-to-understand information presented through diagrams, short videos, or question-and-answer formats. The content should cover psychological support, side effect management, medication consultation, and interpretation of clinical indicators. Participants also valued anonymous peer communication and stepwise information delivery, which reduced the emotional burden.

Sometimes you don’t notice some of our psychological needs. In fact, we are also very anxious and need a lot of psychological support and information.

[P11]

I think the patient communication group is very good. Sometimes I will look at other people’s experiences, and I will refer to them if they are similar to mine. It makes me feel more at ease.

[P6]

There are many side effects of ADT, but there are very few doctors who can really tell me before taking the medicine. They all think it’s not a big problem, but why can’t they issue a manual or column for me to popularize science?

[P9]

### Persona C: Patients Engaging in Family-Doctor–Mediated Information-Seeking

Most of these patients have limited digital literacy and have received a shorter duration of ADT treatment. They have difficulties in understanding and identifying online medical information, so they tend to avoid directly processing complex treatment-related information. They usually have a relatively stable and highly participatory family support system, which can assume the functions of information management and decision-making assistance. Patients mainly play the role of treatment executors, while family members are responsible for screening, interpreting, and transmitting health information.

Motivations: Their main motivation was to reduce the cognitive and practical burden of independently managing complex medical information. Family involvement was viewed as a trusted and efficient way to support treatment management, thereby maintaining role harmony within the family.

My wife has helped me a lot. She will check a lot of information for me. She can even read the test report. She always encourages me to learn about this disease.

[P5]

She remembers what I forgot. If I miss something, she can ask the doctor again (or consult for me online). My daughter knows more than me.

[P20]

Barriers: The main barriers included limited digital skills, difficulty in understanding online medical terminology, and concerns about misinformation or internet scams. Some participants also reported that information became incomplete or simplified during family-mediated communication.

I don’t understand things on the internet, they are trying to teach me kindly. But I can remember some of them, but sometimes the child feels that I don’t understand what he said to me, so he doesn’t tell me.

[P15]

I tried to look it up on my phone, but typing was too slow, and I couldn’t type out many medical terms. After searching for a while, I couldn’t find the information I wanted, so I gave up. It’s more convenient to ask my child.

[P14]

Needs: These patients preferred family-inclusive information support systems that could synchronize reminders, medication schedules, and follow-up information across family members. They also valued clear visual instructions and accessible educational materials that could support understanding when family members were unavailable.

My daughter will help me get medication at the outpatient clinic. If the precautions can be synchronized on a platform or app, I can also see them and won’t worry about forgetting.

[P20]

The child is also very busy sometimes. We only go to the hospital during the follow-up examination. I hope we can also receive professional guidance at home.

[P12]

It would be great to have this care guide, which could include medication introductions, the necessity of certain tests, and what to pay attention to in terms of diet and exercise.

[P5]

### Persona D: Patients Displaying Dependent and Passive Information–Seeking Behavior

These patients generally had limited educational backgrounds, low digital literacy, and minimal active engagement with online health information. Most relied on doctors, family members, or occasional social media exposure for disease-related knowledge. They generally followed medical instructions but lacked a comprehensive understanding of ADT treatment and disease management.

Motivations: Their primary motivation was to maintain daily stability and avoid the burden associated with complex medical information. They tended to trust health care professionals and preferred following instructions rather than independently seeking information.

I can’t understand what the doctor is saying, and I can’t understand the information on the Internet, so I listen to what the doctor asks me to do.

[P3]

The doctors have seen so many patients like me, they must be the most authoritative, and I’m not uncomfortable with the treatment plan he’s given me.

[P18]

Barriers: These patients experienced substantial barriers related to low digital literacy, limited comprehension ability, and concerns about misinformation. Fatalistic beliefs about illness and aging further reduced their willingness to seek information.

Now I have to admit I’m getting old. My mind doesn’t react as quickly as before, and I can’t figure out things like cell phones. Sometimes when I open a website, it jumps to another page, and I don’t dare to click on it casually.

[P18]

As we get older, if we get sick, we treat it. The information on the internet is about the same as what the doctor says, so why not just ask the doctor? There are no other choices...Everyone will experience life, old age, illness, and death.

[P4]

Needs: They preferred low-threshold and easy-to-use health information support focused on practical daily management, including medication guidance, lifestyle advice, follow-up reminders, and reimbursement information. Participants also preferred large fonts, visual or audio materials, and continued access to offline support (face-to-face communication, paper materials). It is necessary to combine text messages and phone reminders to proactively push key information.

I don’t want to know about too complicated content (disease progression, drug selection). But if it’s something experienced in daily life, you can provide it to me, such as diet and exercise. These practical contents are what I care about more.

[P3]

I wish there were a simple guide that tells me the dos and don’ts of treatment. If it is an online platform, I prefer some audio, not too complicated, because it looks difficult to read the words.

[P18]

## Discussion

### Interpretation of the Personas

This study identified 4 personas of OHISB among patients with prostate cancer receiving ADT: proactive information managers, anxiety avoiders, family-mediated seekers, and passive or dependent seekers. Rather than representing fixed patient categories, these personas reflect recurring patterns of information engagement shaped by patients’ emotional responses, treatment experiences, sociocultural context, and perceived ability to manage health information. The findings illustrate the diversity of OHIS experiences during long-term ADT and provide a structured framework for understanding how informational, emotional, social, and contextual factors may interact to influence patients’ engagement with online health information. Recognizing these differing patterns may help inform the development of more responsive and patient-centered information support strategies for men receiving ADT.

Participants classified as proactive information managers demonstrated high levels of disease engagement and digital health literacy, consistent with previous quantitative studies indicating that younger age and higher education are commonly associated with greater information source use among patients with prostate cancer [14,33]. However, the present study further suggests that frequent information-seeking does not necessarily translate into effective information use. Highly engaged patients often assumed substantial responsibility for independently evaluating and integrating complex online information during long-term treatment management, creating a considerable cognitive burden [34]. These findings suggest that improving information organization, source transparency, and navigation support may be more beneficial for this group than simply increasing information availability [10,35]. In clinical practice, health care professionals may help reduce information overload by discussing patients’ online information use during routine follow-up consultations, recommending credible resources, and clarifying misunderstandings. Institutionally endorsed information platforms may also help facilitate patients’ navigation of complex online information during long-term treatment management [36,37].

Anxiety avoiders demonstrated a symptom-triggered and emotionally fluctuating pattern of information engagement, characterized by repeated shifts between intensive searching and abrupt withdrawal. This finding suggests that OHISB may function not only as a cognitive process for acquiring knowledge but also as a strategy for emotional coping under conditions of uncertainty. Previous studies have shown that ADT-related bodily changes, including gynecomastia and sexual dysfunction, may threaten masculine identity, self-image, and autonomy [38]. The present study extends these findings by illustrating how emotional vulnerability may shape patients’ interactions with online information environments. Some participants appeared particularly sensitive to frightening or conflicting information, which intensified uncertainty and contributed to avoidance behaviors [12]. This pattern also reflects the influence of masculine communication norms on OHISB. Consistent with previous evidence that men may avoid direct disclosure of vulnerability and emotional distress, some participants preferred anonymous online communities over discussing sensitive symptoms with clinicians or family members [39]. Within the Chinese cultural context, masculine norms emphasizing emotional restraint and endurance may further reinforce these tendencies [40], highlighting the importance of culturally sensitive information support. Previous studies have also shown that seeking anxiety may delay the collection of treatment information for prostate cancer survivors [41]. Information support for this group may therefore need to address emotional safety in addition to informational needs. Gradual information delivery, emotionally supportive communication, and proactive discussion of sensitive side effects by health care professionals may help patients maintain engagement while reducing distress associated with long-term ADT management [8].

The family-mediated seeker persona highlights the influence of family involvement and health care culture on OHISB within the Chinese context. Previous studies have shown that Chinese families often approach cancer as a collective experience, which may strengthen family cohesion while simultaneously shaping patients’ involvement in health-related decision-making [40]. The present findings suggest that, within family-centered care contexts, engagement with online health information may become a shared and negotiated process rather than an entirely individual activity. In some cases, patients’ access to online information appeared to depend substantially on how family members selected, interpreted, and communicated information during treatment management [42]. Structural characteristics of the Chinese health care system, including limited consultation time, high patient volume, and relatively doctor-centered communication patterns, may further reinforce reliance on family-mediated information support [43]. At the same time, family involvement should not be viewed solely as a barrier to patient autonomy. For many participants, family members represented important sources of emotional reassurance, practical assistance, and treatment coordination. Future digital information support systems may therefore benefit from family-inclusive approaches that engage caregivers while also preserving opportunities for patients to participate directly in the information process through shared platforms, synchronized reminders, and educational resources designed for both patients and caregivers [44].

Passive or dependent seekers in this study appeared to reflect not only limited digital capability but also a preference for minimizing emotional and cognitive burden during long-term illness management. In addition, some participants appeared to view illness progression and aging as largely unavoidable life processes, which reduced the perceived value of actively searching for additional health information. Previous research among Chinese older adults has similarly suggested that beliefs emphasizing acceptance of illness and limited personal control may be associated with lower motivation to seek health information [45]. Although such beliefs may reduce engagement with online resources, they may also represent a culturally and personally meaningful way of maintaining emotional equilibrium during long-term illness management. From this perspective, passive information–seeking behavior should not be interpreted simply as a lack of motivation or disinterest. Rather, for some participants, limiting information engagement appeared to function as an adaptive strategy for maintaining psychological stability in the context of overwhelming or difficult-to-interpret information environments [46]. These findings highlight the importance of respecting patients’ preferred level of information engagement rather than assuming that more information-seeking is always beneficial. Information support for this group may therefore need to prioritize accessibility, clarity, and low-burden communication [9]. Simplified medical language, multimedia educational materials, age-friendly digital interfaces, and sustained availability of offline support channels may help reduce barriers to understandable health information [13].

### Clinical Implications

The findings of this study suggest that OHISB among patients with prostate cancer receiving ADT is shaped by psychological, sociocultural, and capability-related factors. The 4 personas identified in this study may provide implications for practice. In clinical settings, a brief assessment of patients’ preferred information engagement style, digital confidence, emotional responses to health information, and family involvement during routine follow-up consultations may help health care professionals identify patients who require different forms of information support. Rather than uniformly encouraging greater information-seeking, clinicians may need to tailor communication strategies according to patients’ preferred level of engagement, emotional readiness, digital capability, and individual beliefs about illness and personal control. The findings also highlight the importance of culturally sensitive and family-inclusive approaches to information support. Information interventions for patients receiving ADT may benefit from incorporating emotional support, proactive discussion of sensitive side effects, and opportunities for anonymous or low-pressure communication. At the same time, involving family caregivers in information support processes may improve continuity of care while preserving opportunities for patient participation and autonomy. At the digital health system level, future interventions may benefit from combining multiple modes of information delivery, including structured educational platforms, simplified multimedia materials, caregiver-linked systems, and offline support options.

### Limitations

This study has several limitations. First, all participants were recruited from a single tertiary hospital in China, which limits the geographic and institutional transferability of the identified personas. Differences in health care systems, digital health infrastructure, and cultural attitudes across regions may influence how the findings apply to other settings and populations. Second, the personas were constructed from self-reported interview data rather than objective records of actual digital behavior. While qualitative interviews provide the depth and contextual richness necessary for exploratory persona research, future studies combining interview data with platform usage records or digital trace data could offer a more complete account of patients’ information behavior. Third, the 4 personas represent a preliminary qualitative typology derived from a specific clinical context. Larger-scale quantitative and mixed methods studies across diverse populations are needed to further examine the prevalence, stability, and clinical applicability of these personas and to evaluate whether persona-informed information supports improving patient-centered outcomes.

### Conclusions

This study identified 4 personas of OHISB among men with prostate cancer receiving ADT, demonstrating meaningful variation in patients’ motivations, information access preferences, barriers, and support needs. The findings highlight the influence of family-centered care practices and masculine communication norms within the Chinese sociocultural context on how patients engage with online health information. Rather than representing fixed patient categories, these personas provide exploratory and practice-oriented representations of recurring information engagement patterns during long-term ADT management. The findings suggest that future digital health information support may benefit from moving beyond uniform approaches and responding to patients’ diverse information engagement styles through more adaptive, culturally sensitive, and patient-centered strategies. However, further quantitative research is needed to examine the stability, generalizability, and clinical applicability of these personas across different health care settings and populations.

## Supplementary material

10.2196/90567 Multimedia Appendix 1 Semistructured interview guide.

10.2196/90567 Multimedia Appendix 2 Codebook.

10.2196/90567 Multimedia Appendix 3 Matrix of characteristic patterns and persona classifications.

10.2196/90567 Checklist 1 SRQR reporting checklist.

## Abbreviations

ADT: androgen deprivation therapy
OHIS: online health information–seeking
OHISB: online health information–seeking behavior
PSA: prostate-specific antigen
SRQR: Standards for Reporting Qualitative Research
