# Supplementary material for: Tailored Personas of Online Health Information–Seeking Behaviors Among Men With Prostate Cancer Receiving Androgen Deprivation Therapy: Qualitative Study
Source: J Med Internet Res. 2026 Jul 20;28:e90567. doi: 10.2196/90567 (PMC13384350; doi:10.2196/90567)
Supplement: Multimedia Appendix 1 [file jmir-v28-e90567-s001.docx]

| Appendix1 Semi-structured interview guide. |
| --- |
| **Introduction**  Thank you for agreeing to participate in this interview. The purpose of these interviews is to understand the online health information-seeking experiences of patients receiving androgen deprivation therapy, so that we can improve their information support. Please note that there are no right or wrong answers; we are only interested in your thoughts and opinions. Participation in this study is voluntary, and you can exit the study at any time without providing any explanation. The interview will take approximately half an hour to an hour. With your consent, I would like to record the interview because I don't want to miss any of your comments. All answers will be kept confidential. Do you have any questions? May I begin the interview? Therefore, to protect your privacy, we have provided you with a signed confidentiality agreement. I hope you will read it carefully and sign it.  **Initials data**  1. Before starting to share your experiences, I would like to ask you to provide some sociodemographic and clinical data. Could you please tell me:   - Date of birth - Marital status - Educational Level - Current occupational status - Place of residence - Duration of ADT (months) - Current treatments and medication   **Online health information-seeking experience**  2. How long ago were you diagnosed with prostate cancer? How long have you been receiving androgen deprivation therapy?  3. How would you describe your proficiency in using a smartphone or digital devices?  4. How often do you usually seek health-related information online? Under what circumstances do you decide to look for information online?  5. Which platforms or channels do you typically use to find health information related to prostate cancer or androgen deprivation therapy? Why do you prefer these platforms?  (For example, search engines, public health accounts, short videos, patient groups, and medical platforms)  6. Please recall your most recent experience searching online for information related to androgen deprivation therapy, prostate cancer, or treatment-related side effects.  (Tips: What prompted you to search? How did you search? What kind of information did you find?)  7. How would you rate the quality of online information about prostate cancer or androgen deprivation therapy? Was it practically helpful to you?  8. Have you made any adjustments or tried anything based on information found online? For example, changes to lifestyle, exercise, diet, seeking medical care, or how you communicate with your doctor?  (If no—“What do you think prevents you from making changes based on online information?”)  9. What difficulties or challenges have you encountered when searching for health information online?  10. What information related to prostate cancer or androgen deprivation therapy would you most like to find online? Are there topics that are difficult to locate or information you feel is insufficient or unclear?  11. Looking back on your entire androgen deprivation therapy journey, how do you think online health information has influenced or supported your disease management?  12. Overall, how would you describe your experience of seeking health information online during your treatment?  **Thank you very much for your time and the information you shared today.** |
