# Supplementary material for: Tailored Personas of Online Health Information–Seeking Behaviors Among Men With Prostate Cancer Receiving Androgen Deprivation Therapy: Qualitative Study
Source: J Med Internet Res. 2026 Jul 20;28:e90567. doi: 10.2196/90567 (PMC13384350; doi:10.2196/90567)
Supplement: Multimedia Appendix 2 [file jmir-v28-e90567-s002.docx]

| Appendix2. A codebook of online health information-seeking behavior among patients receiving ADT for prostate cancer | | | | |
| --- | --- | --- | --- | --- |
| Category | Subcategory | Code | Definition | Exemplar Quotation |
| 1. Motivation for Seeking Online Health Information | 1.1 Proactive disease management | Seeking treatment rationale and prognosis | Patient searches to understand disease severity, treatment mechanisms, expected duration, and long-term outcomes | *"I want to understand my condition, whether it can be cured, and how to treat it." (P2) "This disease is your own. Health requires you to be proactive, not just let the doctor worry about it." (P15) "After I got the test report, I got a bit nervous and scared, so I would search online to check how my values are and the condition is." (P13) “My wife always asks me to read more knowledge of adt. Only when I understand it can I not panic.” (P5)* |
|  |  | Seeking treatment process clarity | Patient seeks a step-by-step understanding of the treatment trajectory to anticipate what lies ahead | *"I want to figure out the whole treatment process so that I know what will happen next." (P8) “If I can understand the whole procedure, such as why hormone treatment is before or after surgery, and what the long-term side effects are, I can better cooperate with the treatment.” (P19) "Because I've been putting off ADT treatment for over a year, just because I didn't understand the process." (P6)* |
|  | 1.2 Participate in treatment decision-making | Seeking comparative treatment knowledge | Patient seeks side-by-side comparison of treatment options to support informed choice. | *"Various treatment options' benefits and drawbacks — these are what I want to know, rather than simply being told 'we recommend this option'." (P5) “There are so many treatment options for prostate cancer; only when I understand all of them can I know which one is suitable for me now and make the best choice,you know AI can help me organize this.” (P1)* |
|  |  | Seeking medication and treatment updates | Patient actively seeks information about new drugs or protocol changes to maintain decisional agency | *"Medications for ADT treatment keep coming out with new ones, and have been switched many times. I can learn a bit more and then make a decision." (P9) “I've joined a patient online group that has treatment guidelines, and I just read them and realized that ADT treatments are now evolving so fast too. It used to be whatever the doctor said to use, but now I know myself what new endocrine treatments are probably out there.”(P10) “Medications are updated quickly, and I often switch Medications . Without understanding, it can be confusing.”(P16)* |
|  | 1.3 Symptom-driven seeking | Distinguishing side effects from disease progression | Patient searches online to determine whether a new bodily change is a drug reaction or disease worsening | *“After switching to a new medication, I experienced muscle soreness and joint pain. I’m not sure whether it’s side effects or disease progression. I have been looking for ways to alleviate it.” (P9) “I sweated at night, my mouth was dry, and my back hurt. I don't know if it was metastasis or a side effect, so I went to the QQ group to ask, and the patient said it was a medication reaction.” (P11) “If I'm not feeling well, I'll ask my daughter to check it for me. Basically, I can get the answer online.”(P12) “I'm not a local, so it's very troublesome to register. If there are (symptoms), I'll check them online. If you compare them more, you'll probably know it's a side effect.”(P4)* |
|  |  | Seeking practical symptom management | Patient searches for specific, actionable methods to alleviate physical symptoms of ADT | *"The most troubling thing is hot flashes. Sometimes I break out in a sweat just sitting there. I want to know what methods can relieve it." (P16) “I got a lot of rashes when I took apatamine. At this time, I thought of searching for information, because the doctor just told me that it might happen, but I didn't know it was so serious.”(P6) “Actually, I don't like to look up things on the Internet. I usually only check it when I feel unwell.”(P3)* |
|  | 1.4 Uncertainty and anxiety reduction | Peer normalisation and validation | Patient seeks others' experiences to validate own symptoms as normal and reduce isolation | *"Patients in the group always share their experiences with each other, which has also made me more actively involved to some extent." (P2) “My local friend also received ADT treatment and I was definitely anxious and reached out to him for counseling. It's only informative if the experience is similar." (P18)* |
|  |  | Alleviating anxiety through information | Patient searches online to reduce fear or uncertainty about unknown aspects of their condition or treatment | *“For me, knowing more information can make me feel more at ease.”(P10) “When you feel uncomfortable, it's you who want to know why and how to solve it, otherwise who will check it on the Internet?” (P17) “Sometimes not understanding is also a way to exacerbate anxiety......” (P6)* |
|  |  | Bridging outpatient communication gaps | Patient seeks online information to supplement insufficient time or detail provided during clinic visits | *"The time in the outpatient clinic is too short. Many questions cannot be asked, so I have to search online myself." (P13) "The general outpatient clinic is like an assembly line."(P7) “The doctor just tells me to do examinations, get injections, or take medicine without explaining why.” (P19)* |
|  | 1.5 Identity and role maintenance | Maintaining sense of control through information | Patient proactively seeks information as a means of preserving personal agency, self-efficacy, and active participation in treatment decisions | *“Obtaining more information makes me feel secure in many aspects, including psychologically, because I will not have doubts in my mind. Understanding sufficient information gives me a sense of certainty.” (P1) "I want to be involved in my own treatment decisions. The doctor recommends, but I need to understand why." (P7) "I have been tracking my PSA changes myself, knowing the numbers helps me feel in control." (P8)* |
|  |  | Selective information seeking to manage identity threat | Patient strategically limits or filters information to protect psychological wellbeing and sense of self in the face of ADT-induced bodily changes. | *"I don't want to know too much about things that cannot be changed, it only makes me feel worse." (P18) "Some things I prefer not to look up. Knowing will not help; it will only add to my worries." (P4) “After all, as a man, I think it's still most important to know one's own condition. Let others check it? No, better to check when there's a problem.” (P17) “I'm more vulnerable, so I don't want to pay too much attention to this matter (prostate cancer)” (P20)* |
|  | 1.6 Passive or avoidant orientation | Relying on medical authority | Patient defers information-seeking to healthcare providers, trusting their expertise over self-directed online search. | *"I can't understand what the doctor is saying, and I can't understand the information on the Internet, so I listen to what the doctor asks me to do." (P3) “The doctors have seen so many patients like me, they must be the most authoritative, and I'm not uncomfortable with the treatment plan he’s given me.” (P18)* |
|  |  | Avoiding information burden | Patient deliberately limits information-seeking to prevent anxiety or disruption to daily life. | *"I don't want to know about too complicated content." (P3) "I prefer not to look at too much online. The more you read, the more worried you become." (P4) “I'm a local, and I'm also familiar with xx hospital. I've been seeing this doctor for many years. His information is useful enough, and it is also a burden to read too much on the Internet.” (P14) “I don't really want to give this (prostate cancer) too much attention, I'll read it when it's pushed to, but I won't check it out either. Can't be too concerned if you need to stay in a good frame of mind.”(P18)* |
| 2. Online Information Access Preferences | 2.1 Institutional and physician-endorsed sources | Trusting physician-endorsed online platforms | Patient prefers or seeks online platforms explicitly recommended or verified by healthcare providers. | *“In my opinion, if someone wants to get information from the Internet, it is at least the responsibility of hospitals to recommend authoritative websites to patients to make sure they know what is right and what is wrong.”(P13) “Some doctors recommend some resources to me, and I will study them carefully.”(P19) “I've been to other hospitals before. The doctors there have online consultation, and he will also see a doctor on other platforms. It's more convenient for people from other places like us.”(P4)* |
|  |  | Using medical consultation platforms | Patient uses structured physician-facing platforms (e.g., Haodf) for direct medical queries. | *"I sometimes ask questions on the Haodf platform." (P6) "This platform lets me consult specialists directly — that is much more reliable than random websites.” (P14) "Compared to other websites, doctors on professional medical platforms are named, making them very trustworthy." (P20)* |
|  | 2.2 Non-institutional digital platforms | Using general internet and social media platforms | Patient uses Baidu, TikTok (Douyin), Xiaohongshu, or general websites for disease information, without institutional endorsement. | *"I search on Xiaohongshu, and some doctors on Douyin also do popular science. It is not harmful to check often." (P1) “Of course, I'll check Baidu online, I need to get an idea of it, find out what kinds of needles and medicines there are.”(P7) “Sometimes I will read the posts written by people with diseases like us on Xiaohongshu, encourage each other, and learn from experience.”(P12)* |
|  |  | Engaging in online patient communities | Patient actively participates in peer patient groups for experiential knowledge, emotional support, and symptom normalisation. | *"I joined a patient group with several thousand people. The questions and answers are very detailed. I really like it." (P9) "I will communicate with other patients, see if there's anything similar to my situation." (P12) “The QQ patient group is really good. People will share information and asso questions in the group.”(P11)* |
|  | 2.3 Family-mediated access | Family as online information proxy | Family members search, filter, or explain online health information on the patient's behalf, with the patient as a passive recipient. | *"My wife has helped me a lot. She will check a lot of information for me. She can even read the test report." (P5) "She remembers what I forgot. I just tell her my symptoms and she looks it up." (P20)* |
|  |  | Family as interpreter and communication bridge | Family members accompany patients to consultations, translate medical information into lay terms, and relay questions to providers on the patient's behalf. | *"My child always comes with me. They ask the questions I forget, and explains what the doctor said afterwards." (P14) "My son looked it up and then explained it to me in simple words." (P15) “My legs are not very convenient, and my sister usually help me get the medicine. I usually ask her if there are any issues.” (P3)* |
| 3. Barriers to Online Health Information Seeking | 3.1 Cognitive and health literacy barriers | Difficulty understanding medical terminology | Patient cannot comprehend medical terminology, complex explanations, or professional jargon encountered online. | *"I do not understand this disease. I do not know how to ask. Sometimes I cannot understand what you say." (P3) “I usually don't comment because I don't understand it either; I just refer to it and take a quick look.”(P15) “I tried to look it up on my phone, but typing was too slow, and I couldn't type out many medical terms. After searching for a while, I couldn't find the information I wanted, so I gave up. It's more convenient to ask my child. ”(P14)* |
|  |  | Overwhelmed by information volume and contradictions | Patient is distressed by the abundance, inconsistency, or poor quality of online health information, reducing trust and usability. | *“The information on the Internet is too scattered, and there are too many popular science accounts on TikTok and Xiaohongshu now. I can't be sure which one is right.”(P2) “There are different statements in different places. For example, it is difficult to judge the long-term impact of side effects.” (P1) "There is too much information online, some statements are completely opposite; I do not know which to believe. Some websites are obviously selling products." (P4)* |
|  | 3.2 Digital skill barriers | Limited digital navigation skills | Patient lacks ability to use smartphones, apps, or search engines effectively for health information seeking. | *“I don’t understand things on the internet, they are trying to teach me kindly. But I can remember some of them, but sometimes the child feels that I don’t understand what he said to me, so he doesn’t tell me.” (P15) “Now I have to admit I’m getting old. My mind doesn’t react as quickly as before, and I can’t figure out things like cell phones. Sometimes when I open a website, it jumps to another page, and I don’t dare to click on it casually.” (P18)* |
|  |  | Fear of online risks | Patient avoids online searching due to concerns about scams, misinformation, or device security. | *"I do not dare click randomly; the phone might get a virus. In WeChat groups, I basically just read what they send." (P20) “The doctor said not to believe too much on the Internet, because there are so many problems.”(P10) “I have bought things online before and been scammed out of a lot of money.”(P8)* |
|  | 3.3 Emotional and psychological barriers | Fear-induced information avoidance | Patient stops or avoids searching because online content triggers fear, anxiety, or catastrophic thinking. | *“The language on the Internet, you know, they write very scary in order to attract attention, and I don’t dare to look down when I see it. What ‘beware of these symptoms’ and ‘late signals’, I didn't dare to look down when I saw the title.” (P16) "The more I read, the more scared I become, so I just stop." (P7) “I won't read it if I know about it. I feel that it's not good to know too much. It looks scary, doesn't it?”(P17)* |
|  |  | Shame-driven avoidance of sensitive health information | Patient avoids seeking, disclosing, or discussing sensitive health topics related to ADT-induced bodily changes (e.g., sexual dysfunction, gynaecomastia) due to shame, embarrassment, or fear of social judgment. This barrier operates both in online information seeking and in patient–provider communication. | *“I do worry... my breasts have grown bigger. It’s actually a burden for me. But you won’t ask me, so I won’t mention it on my own.” (P17) "I feel embarrassed about these changes. I would rather not bring it up — even with the doctor." (P6)* |
|  |  | Fatalistic beliefs reducing information motivation | Patient believes seeking more information will not change outcomes, reducing motivation to engage in online health information seeking. | *“As we get older, if we get sick, we treat it. The information on the internet is about the same as what the doctor says, so why not just ask the doctor? There are no other choices... Everyone will experience life, old age, illness, and death.” (P4) "Whatever happens, happens. Reading more about it will not change anything." (P3)* |
| 4. Unmet Information Needs | 4.1 Content needs | Structured, stage-specific treatment information | Patient desires a well-organised, stage-by-stage resource covering treatment processes and anticipated side effects. | *“If the common problems can be sorted out by category, I don’t have to piece them together.” (P7) “Some classified knowledge content may be more in line with the needs of patients.” (P2) “I may prefer the knowledge presented by classification, just like the xx official account. The column below it is different (classification). You can see what you are interested in.” (P1)* |
|  |  | Symptom management guidance | Need for specific, actionable guidance on managing ADT side effects at home. | *“There are many side effects of ADT, but there are very few doctors who can really tell me before taking the medicine. They all think it's not a big problem, but why can't they issue a manual or column for me to popularize science?” (P9) “It would be best to tell us different priorities at different stages, such as explaining what changes might occur before taking medication, how to relieve them, and later discussing possible subsequent treatments.” (P12) “... and I want to know what is the best way to deal with muscle soreness and mild joint pain." (P8)* |
|  |  | Lifestyle and dietary self-management | Need for diet, exercise, sleep, and daily living recommendations tailored to ADT patients. | *"Regarding diet, which foods can be eaten, which cannot; which are bad for my disease, which are beneficial for recovery?" (P18) “It would be great to have this care guide, which could include medication introductions, the necessity of certain tests, and what to pay attention to in terms of diet and exercise.” (P5) “I don't want to know about too complicated content (disease progression, drug selection). But if it's something experienced in daily life, you can provide it to me, such as diet and exercise. These practical contents are what I care about more.” (P3)* |
|  |  | Medication education | Need for clear, patient-accessible information about medication names, dosing schedules, and consequences of missed doses. | *“I need a manual about the medicine and side effects.” (P10) "... When you are old, it is easy to forget. If you don't take the medicine on time or miss the medicine, will it have a big impact? Sometimes I really need to be reminded to remember" (P12) “I wish there were follow-up and medication reminders”. (P6)* |
|  |  | Psychological coping guidance | Need for professional guidance on mood regulation, anxiety management, and emotional adaptation to ADT. | *“Sometimes you don’t notice some of our psychological needs. In fact, we are also very anxious and need a lot of psychological support and information.” (P11) "My mood swings are sometimes severe. If you could teach us some methods to regulate emotions, that would be even better." (P16)* |
|  | 4.2 Format and delivery needs | Multimodal, accessible formats | Patient needs non-text or mixed-media formats (audio, video, illustrated guides, large font) due to reading difficulty, low literacy, or visual preference. | *“I wish there were a simple guide that tells me the dos and don’ts of treatment. If it is an online platform, I prefer some audio, not too complicated, because it looks difficult to read the words.” (P18) “I'd rather watch a 5-min video than read a book...Don't talk (professionally) and don't understand. It's enough to be useful in daily life.” (P4) "I prefer the form of images and text together, so I can be more interested in reading it. Also, do not pile a lot of text and information together; looking at it makes my eyes dizzy and I cannot understand." (P13)* |
|  |  | Emotionally sensitive content presentation | Patient prefers information that is empathetic, non-alarmist, and gently framed to avoid triggering anxiety or avoidance. | *"The more you look into it, the more it affects your mood. So be careful with your language." (P16) "If the online information could be gentle and not too shocking, I would be more willing to read it." (P7)* |
|  | 4.3 Support and interaction needs | Structured peer support access | Need for organised access to other patients' real experiences for emotional validation and practical reference. | *"I think the patient communication group is very good. I will look at other people's experiences. It makes me feel more at ease." (P6) "I think we should communicate more with patients, because you can learn about the situation similar to yours between patients, which medicine is the most effective and whether it is good or not."(P2)* |
|  |  | Shared family information platform | Need for family members to access synchronised information, schedules, and medication reminders alongside the patient. | *“My daughter will help me get medication at the outpatient clinic. If the precautions can be synchronized on a platform or app, I can also see them and won’t worry about forgetting.” (P20) “The child is also very busy sometimes. We only go to the hospital during the follow-up examination. I hope we can also receive professional guidance at home.” (P12)* |
|  |  | Direct physician communication channel | Need for digital access to healthcare providers for ongoing, asynchronous consultation. | *“It's hard to see a doctor now. I wish I could communicate with the doctor on WeChat. It's better to record my PSA level and the symptoms after taking the medicine.” (P2) “I think developing a WeChat Mini Program would be the best option. The backend can also view our information and requirements.” (P8) “You can set up a message area. If we have any questions, we can leave a message. It's also good for you to reply when you are free.” (P19)* |
