# Supplementary material for: Tailored Personas of Online Health Information–Seeking Behaviors Among Men With Prostate Cancer Receiving Androgen Deprivation Therapy: Qualitative Study
Source: J Med Internet Res. 2026 Jul 20;28:e90567. doi: 10.2196/90567 (PMC13384350; doi:10.2196/90567)
Supplement: Multimedia Appendix 3 [file jmir-v28-e90567-s003.docx]

| **Appendix3: Matrix of characteristic patterns and persona classifications** | | | | | |  |  |
| --- | --- | --- | --- | --- | --- | --- | --- |
| Persona Type | P | Motivation | Information Access Preferences | Barriers | Information Support Needs | Digital literacy | Seeking frequency |
| Proactive Information Manager (n=6) | P1 | Seeking comparative treatment knowledge; maintaining sense of control through information | General internet platforms; physician-endorsed platforms | Information overload; contradictory content | Structured stage-specific information; direct physician communication | ① | ① |
|  | P2 | Seeking treatment rationale and prognosis; peer validation | General internet platforms; online patient communities | Information fragmentation; inconsistent quality | Direct physician communication channel; structured peer support | ② | ② |
|  | P7 | Seeking treatment process clarity; bridging outpatient communication gaps | General internet platforms; physician-endorsed platforms | Information inconsistency; distress from negative content | Structured stage-specific information; emotionally neutral content | ② | ① |
|  | P8 | Seeking treatment process clarity; maintaining sense of control through information | General internet platforms; online patient communities | Information overload; contradictory content, online fees expensive | Symptom management guidance; structured treatment information | ② | ① |
|  | P13 | Seeking treatment rationale; supplementing outpatient information | Physician-endorsed platforms; medical consultation platforms | Limited consultation time; information fragmentation | Structured information with visuals; institutionally verified platforms | ① | ① |
|  | P19 | Seeking treatment process clarity; supplementing clinical explanations | Physician-endorsed platforms; direct physician communication | Insufficient authoritative and easily understandable information on the internet | Pre-treatment ADT education; stage-specific explanations | ② | ② |
| Anxiety Avoider (n=6) | P6 | Symptom-driven seeking (rash from apatamide); peer normalization; uncertainty reduction (treatment delayed >1 year due to unclear process) | Medical consultation platform (Haodf); online patient communities | Shame-driven avoidance of gynaecomastia-related symptoms; reluctance to disclose to provider | Structured peer support; medication reminders and follow-up; sensitive symptom information | ③ | ② |
|  | P9 | Symptom-driven seeking (side effects vs. disease progression); seeking medication updates due to frequent switches | Online patient communities; active medication update monitoring across platforms | Fear-induced avoidance of alarming online content (sensationalized headlines) | Pre-treatment ADT side-effect manual; psychological coping guidance; empathetic non-alarmist content | ② | ② |
|  | P10 | Alleviating anxiety through information; seeking medication and treatment updates via patient groups | Online patient groups with treatment guidelines; online patient communities | Fear of online risks and misinformation (doctor-advised scepticism); avoidance when content is alarming | Medication education; anxiety self-management tools; reliable curated information | ③ | ② |
|  | P11 | Symptom-driven seeking (night sweats, back pain — metastasis vs. side effect?); psychological support | QQ patient group; active peer community participation for symptom normalization | Perceived invisibility of emotional distress among providers | Psychological coping guidance; structured peer support network; emotional validation resources | ③ | ② |
|  | P16 | Seeking practical symptom management (hot flashes); seeking medication updates | General internet search; social media science content (Douyin); emotionally cautious browsing | Fear-induced information avoidance triggered by alarming headlines and scary framing | Psychological coping and emotion regulation guidance; emotionally sensitive, non-shocking content | ④ | ③ |
|  | P17 | Selective information seeking to manage identity threat; symptom-driven but shame-limited | Private, selective online search; highly reluctant to disclose symptoms to others or providers | Shame-driven avoidance (sexual dysfunction, gynaecomastia); fear-induced avoidance of sensitive topics | Discreet, non-judgmental information on ADT-induced physical changes; sensitive content framing | ② | ② |
| Family-Mediated Seeker (n=5) | P5 | Treatment decision-making support; proactive disease management (primarily wife-driven) | Family as online information proxy (wife manages search, interprets reports, tracks information) | Reliance on spouse; patient takes passive role in information-seeking | Shared family care platform; lifestyle and dietary self-management guide for patient and family | ③ | ③ |
|  | P12 | Symptom-driven seeking (family mediated); maintaining care continuity; stage-specific information | Family as information proxy (daughter retrieves medication and information); general internet search by family | Difficulty understanding medical terminology; care gaps when family is unavailable | Symptom management guidance; shared family information platform; home care professional support | ③ | ③ |
|  | P14 | Reducing cognitive and physical burden of search | Family as interpreter and communication bridge (child accompanies consultations, types queries) | Limited digital navigation skills (cannot type medical terms); difficulty understanding terminology | Simplified family-mediated digital resources; pictorial instructions; family-synchronized reminders | ② | ③ |
|  | P15 | Seeking treatment rationale passively through family; limited self-initiated motivation | Family as interpreter (son explains in lay terms); passive recipient of family-filtered information | Limited digital navigation skills; information loss and breakdown in family transmission chain | Clear, simple content for independent reference; pictorial guides; intergenerational communication support | ③ | ③ |
|  | P20 | Passive orientation with family mediation; fear of online risks limits independent search | Family as online information proxy (daughter manages appointments, medication, and information) | Fear of online risks (previous online scam experience); passive digital engagement | Shared family information platform (synchronized app); daughter-patient medication reminders | ③ | ② |
| Passive-Dependent Seeker (n=3) | P3 | Passive/avoidant orientation; symptom-driven seeking only when unwell; relying on medical authority | Family as interpreter (sister mediates); minimal and reluctant independent search | Difficulty understanding medical terminology; low digital literacy; information burden avoidance | Dietary and lifestyle guidance; simplified audio content; family-assisted access to basic information | ④ | ③ |
|  | P4 | Fatalistic beliefs reducing information motivation; physician authority deference; symptom-driven when necessary | Physician-endorsed online consultation when symptomatic and away from home; otherwise avoidant | Fatalistic beliefs ('everyone experiences illness and death'); information overload aversion | Simplified multimodal content (audio, video); dietary guidance; low-complexity health education | ③ | ④ |
|  | P18 | Passive/avoidant orientation; avoiding information burden; relying entirely on medical authority | Passive receipt of physician instructions only; strong preference for audio over text; avoids independent search | Limited digital navigation skills (technology anxiety, accidental page jumps); information burden avoidance | Simple audio guide (diet, exercise, medication dos and don'ts); low-threshold digital access; large-font content | ④ | ④ |

Note. Participant profiles summarize coded characteristics across all meaning units; they do not reproduce original quotes in full. Full exemplar quotations with participant codes are provided in the codebook (Appendix 2).¹ Digital literacy (smartphone proficiency): ① Very proficient (can handle various tasks); ② Moderately proficient (frequent use of social media, shopping, news apps); ③ Basic (only basic functions such as WeChat chat and video watching); ④ Not proficient, needs assistance.² Seeking frequency (frequency of seeking disease‑related health information): ① Always (several times per day); ② Often (several times per week); ③ Occasionally (several times per month); ④ Never.
